# Parents' psychological adjustment in families of children with Spina Bifida: a meta-analysis

**DOI:** 10.1186/1471-2431-5-32

**Published:** 2005-08-25

**Authors:** Ignace PR Vermaes, Jan MAM Janssens, Anna MT Bosman, Jan RM Gerris

**Affiliations:** 1 Institute of Family and Child Care Studies, Radboud University Nijmegen, Montessorilaan 3, P.O. Box 9104, 6500 HE Nijmegen, The Netherlands; 2 Department of Educational Sciences, Radboud University Nijmegen, Montessorilaan 3, P.O. Box 9104, 6500 HE Nijmegen, The Netherlands

## Abstract

**Background:**

Spina Bifida (SB) is the second most common birth defect worldwide. Since the chances of survival in children with severe SB-forms have increased, medical care has shifted its emphasis from life-saving interventions to fostering the quality of life for these children and their families. Little is known, however, about the impact of SB on family adjustment. Reviewers have struggled to synthesize the few contradictory studies available. In this systematic review a new attempt was made to summarize the findings by using meta-analysis and by delimiting the scope of review to one concept of family adjustment: Parents' psychological adjustment. The questions addressed were: (a) do parents of children with SB have more psychological distress than controls? (b) do mothers and fathers differ? and (c) which factors correlate with variations in psychological adjustment?

**Methods:**

PsycInfo, Medline, and reference lists were scanned. Thirty-three relevant studies were identified of which 15 were eligible for meta-analysis.

**Results:**

SB had a negative medium-large effect on parents' psychological adjustment. The effect was more heterogeneous for mothers than for fathers. In the reviewed studies child factors (age, conduct problems, emotional problems, and mental retardation), parent factors (SES, hope, appraised stress, coping, and parenting competence), family factors (family income, partner relationship, and family climate), and environmental factors (social support) were found to be associated with variations in parents' psychological adjustment.

**Conclusion:**

Meta-analysis proved to be helpful in organizing studies. Clinical implications indicate a need to be especially alert to psychological suffering in mothers of children with SB. Future research should increase sample sizes through multi-center collaborations.

## Background

Worldwide, SB is the second most common congenital birth defect [1]. Its prevalence varies per geographic region, depending on genetic and environmental factors [2]. In developing countries the occurrence of SB tends to be higher than in Western countries. For example, in Tanzania the incidence of SB live births is estimated at 1.35‰ [3] whereas in the US, the incidence of SB pregnancies is estimated at 0.41‰ [4] and the number of SB live births at 0.21‰ [5]. Thus, despite primary prevention programs, such as the fortification of cereal grain products with folic acid in the US [5], and despite estimates that at least 40% of the early detected SB pregnancies in Europe are terminated [6], the number of children who are born with SB remains substantial.

Children with spina bifida (SB) live with a range of disabilities, depending on where in the spinal column formation the defect is located and whether it is closed or open. Since the mid 1960s, early surgical treatment of SB has increased the survival rates of children with severe forms of SB and in more recent years, the development of prenatal surgery around the 20^th ^week of pregnancy has further improved children's chances of survival [7]. Consequently, medical teams face the task of fostering the quality of life for these children and their families. On the one hand, enhancing the quality of life depends on medical advances (e.g. urological, orthopedic, and hydrocephalus research). On the other hand, it depends on the development of a scientifically based understanding of the psychosocial aspects involved with chronic illness in general and SB in particular [8].

To date, a limited number of studies have investigated psychosocial aspects of SB. Typically, these studies have focused on two broad topics: (1) the impact of SB on the child and (2) the impact of SB on the family [8]. Although attempts have been made to integrate findings [8-12], most reviewers have struggled to draw conclusions on family adjustment to SB. One problem is the dearth of empirically sound studies. Another problem is the small number of studies with theoretically driven research questions and hypotheses [8]. Both problems have led to a fragmented picture of mixed findings, because the few studies available have investigated outcome variables reflecting different levels of family functioning (e.g. marital adjustment, parenting stress, and family atmosphere) as indicators of family adjustment. Based on family-systems theory and family-resilience theory it can be argued that SB will have a differential effect on different levels of the family structure [13].

Therefore, in this review a new attempt was made to synthesize findings by concentrating on one level of family adjustment only: parental adjustment. Moreover, the traditional narrative methods used by earlier reviews were replaced with statistical meta-analysis to summarize findings more systematically. The goal of this approach was to exhaust the limited studies available to maximize the information concerning parents' adjustment to having a child with SB.

### Conceptualization of adjustment: psychological adjustment

A preliminary inventory of the literature uncovered that we could divide the research on parental adjustment to SB into three areas: (1) psychological adjustment, (2) interpersonal adjustment in the dyadic partner and parenting relationships, and (3) parents' perceptions of the family atmosphere. The inspiration to discern these areas of adjustment stemmed from Wallander's model of maternal adjustment with chronic illness [14]. In this model the areas mental health, physical health and social functioning are distinguished as indicators of maternal adjustment.

Parents' psychological adjustment can be defined as the adaptive task of managing upsetting feelings aroused by the illness of the child and preserving a reasonable emotional balance [15]. Pless and Pinkerton [16] have postulated that adjustment to chronic illness changes over time and that at any given moment psychological adjustment will reflect the cumulative product of earlier transactions. Thus, on the one hand parents' psychological adjustment reflects the outcome of parents' ability to maintain a balance between the demands of stressful situations and the availability of personal (e.g. optimism) and social resources (e.g. partner support), whereas on the other hand, parents' psychological adjustment enhances the accomplishment of other general adaptive tasks, such as: preserving a satisfactory self-image, keeping the family together, and preparing for an uncertain future, as well as the accomplishment of illness-related tasks, for example: dealing with the symptoms of the illness, dealing with treatment related stressors, and establishing functional relationships with health caregivers [15]. Positive experiences in achieving such tasks will in turn enforce parents' emotional balance through so called positive-feedback loops [16].

Based on these ideas, we opted to delimit the concept of parental adjustment to parents' psychological adjustment. Only studies using psychological outcomes as indicators of parents' adjustment to SB were included in the review.

### Hypotheses and research questions

A considerable number of studies have been devoted to children with severe disabilities and their families [17]. Two approaches have emerged: A categorical and a non-categorical. Categorical studies aim at investigating the unique effects of a specific disease on family life, for example SB, whereas non-categorical studies aim at examining shared effects of different chronic diseases on family life [18].

From non-categorical accounts a few broad hypotheses regarding parents' psychological adjustment with SB were derived. Parents of children with physical impairments have been found to report higher levels of stress, anxiety, and depression than parents of able-bodied children [19], however parents' adjustment to chronic illness has also been found to be marked by great individual variation [14,16]. Studies have yielded several conceptual models based on stress-coping theories and socio-ecological views on family functioning to explain the differential effects of chronic illness on parents' adjustment [19]. In short, most of these models view the child's chronic illness as a potential stressor. The severity of the illness and associated delays in the child's development are expected to determine the functional care strain on the family as a whole and on parents in particular. Besides illness-related stressors, other major life events and daily hassles may add to the demands on parents. Stress-coping theories maintain that the extent to which parents are negatively affected by these demands, will depend on how they appraise, or give meaning, to them. In the process of appraisal parents estimate how their personal capacities and their resources of social support meet the demands of stressful situations. The personal capacities to interact with stressful situations are determined by parents' personality characteristics, coping styles, and strategies. The social resources are determined by the extent to which parents have access to emotional as well as instrumental support from their relationships with others, for example, marital support, family support, informal support from extended family and friends, and formal support from professional caregivers. Depending on how parents estimate the balance between the illness-related stressors, their personal capacities, and their social resources, they can be expected to have more or less difficulties to adjust to having a child with SB. Thus, variability in parents' psychological adjustment can be expected to be associated with multiple factors concerning: characteristics of the child (e.g. severity of illness and developmental delays), characteristics of the parent (e.g. personality characteristics and coping styles), characteristics of the family (e.g. marital quality and family climate), and characteristics of parents' environment outside the family (e.g. social support from extended family and friends).

Although most studies have focused on maternal adjustment to chronic illness, individual differences may be expected between mothers and fathers because of role differentiations in care and work [19]. Mothers are often their child's main caregiver. Consequently, they are more exposed to illness-related situations than fathers and may therefore experience more psychological stress than fathers.

In this review the above premises were studied, guided by three research questions identified in the literature on parents' adjustment with SB: (a) do parents of children with SB have higher levels of psychological distress than do parents of able-bodied children? (b) do mothers and fathers differ in psychological adjustment? and (c) which factors are correlated with parents' psychological adjustment? Four categories of factors were discerned: (a) child factors, (b) parent factors, (c) family factors, and (d) other environmental factors.

## Methods

### Identification of studies

For the meta-analysis, primary research reports were located and coded in four steps:

#### Step 1: Identification of studies on parents' adjustment

The PsycInfo and Medline databases from 1966 to January 2005 were scanned using the key terms "spina bifida" or "neural tube defect" (NTD) or "myelomeningocele" (MMC) and "family" or "parenting" or "parents" and "adjustment" or "adaptation". This resulted in 925 abstracts. Two reviewers (IV and JJ) selected 65 abstracts based on the following inclusion criteria: (1) available in English, (2) reported primary research, and (3) studied parents' adjustment with SB. Agreement between raters was 96.6% (Cohen's Kappa = .92). Differences between reviewers were resolved through discussion.

The reference lists of the 65 reports were scanned to check whether other studies had been missed in the first scan of PsycInfo and Medline. Despite this check, one report was overlooked because at first glance its appearance was similar to another report of the same authors published in the same year [20,21].

#### Step 2: Selection of studies on parents' psychological adjustment

The two reviewers coded each publication with regard to the area of parents' adjustment. Three areas were distinguished: (1) individual psychological adjustment, (2) interpersonal adjustment in dyadic partner and parent-child relationships, and (3) perceptions of family functioning. The coders found that 33 out of 66 studies reported findings on psychological adjustment. Their interrater reliability was 90.8% (Cohen's Kappa = .82). Total agreement was achieved through discussion.

#### Step 3: Coding of research reports

The 33 studies were classified by study and sample characteristics (see Table 1). The study characteristics were: number of participants, design, presence of comparison group, and outcome measure. The sample characteristics were: parent gender, child impairment, child age, and treatment timing of SB. The two coders agreed between 87% and 100% (Cohen's Kappa = .84 to 1.00). Discussion led to total agreement.

**Table 1:** Study and sample characteristics of reports on parents' psychological adjustment

**Reports included in meta-analysis**	***N***	**Design**	**Comparison Group**	**Parent Gender**	**Child Impairment^1^**	**Child Age**	**Child Treatment**	**Outcome Measure^2^**
Barakat & Linney, 1992 [31]	29	Prospective	Control	Mothers	SB (MMC-non retarded)	6–11	Early	BSI
Barakat & Linney, 1995 [32]	29	Prospective	Control	Mothers	SB (MMC-non retarded)	6–11	Early	BSI
Evans, Tew, & Laurence, 1986 [48]	124	Longitudinal	Control	Fathers	Combined: NTD	18	Late	General Health Questionnaire
Fagan & Schor, 1993 [61]	50	Prospective	Norm scores	Mothers	SB	M = 8.1	Early	Malaise Inventory
Holmbeck, Gorey-Ferguson, Hudson, Seefeldt, Shapera, Turner, & Uhler, 1997 [43]	55	Prospective	Control	Mothers & fathers	SB	8–9	Early	SCL-90R
Horton & Wallander, 2001 [23]	33	Prospective	Norm scores	Mothers	SB	M = 10.6	Early	BSI
Kazak & Marvin, 1984 [62]	56	Prospective	Control	Mothers & fathers	SB (MMC)	1–16	Early	Langner Symptom Checklist
King, King, Rosenbaum, & Goffin, 1999 [22]	164	Prospective	Norm scores	Mothers & fathers	Combined: CP, SB, NOS	3–6	Early	SCL-90R
Kronenberger & Thompson, 1992 [20]	66	Prospective	Norm scores	Mothers	SB (MMC)	0–18	Early	SCL-90R
Kronenberger & Thompson, 1992 [21]	66	Prospective	Norm scores	Mothers	SB (MMC)	0–18	Early	SCL-90R
Lemanek, Jones, & Lieberman, 2000 [56]	59	Prospective	Norm scores	Mothers	SB-non retarded	3–16	Early	SCL-90R
Tew & Laurence, 1973 [33]	51	Longitudinal	Norm scores	Mothers	SB	M = 11.6	Late	Malaise Inventory
Tew & Laurence, 1975 [34]	51	Longitudinal	None	Mothers	SB	M = 11.6	Late	Malaise Inventory
Wallander, Varni, Babani, DeHaan, Thompson, Wilcox, & Tweddle Banis, 1989 [14]	50	Prospective	Norm scores	Mothers	Combined: SB, CP	6–11	Early	Malaise Inventory
Wiegner & Donders, 2000 [45]	34	Prospective	Norm scores	Mothers	SB	3–12	Early	BSI

**Reports excluded from meta-analysis**	***N***	**Design**	**Comparison Group**	**Parent Gender**	**Child Impairment^1^**	**Child Age**	**Child Treatment**	**Outcome Measure^2^**

Dorner, 1973 [63]	63	Prospective	None	Mothers	SB	13–19	Late	Malaise Inventory
Dorner, 1974 [44]	63	Prospective	None	Mothers	SB	13–19	Late	Malaise Inventory
Dorner, 1975 [64]	63	Prospective	None	Mothers	SB	13–19	Late	Malaise Inventory
Dorner & Atwell, 1985 [65]	25	Prospective	None	Mothers & fathers	Non-surviving SB	-	-	Malaise Inventory
Downey, 1981 [66]		Cohorts	None	-	Combined: SB, Down syndrome	0–2	-	Standardized questionnaire
Eden-Piercy, Blacher, & Eyman, 1986 [67]	77	Prospective	None	Mothers	Combined: SB, autistism, mentally retarded	1–10	Early	Questionnaire on emotions
Hare, Laurence, Payne, & Rawnsley, 1966 [52]	120	Longitudinal	None	Mothers & fathers	Combined: SB, ANC, HYDRO		Late	Semi-structured interview
Kazak, 1987 [46]	125	Prospective	Control	Mothers & fathers	Combined: SB, PKU, mentally retarded	1–16	Early	Langner Symptom Checklist
Kolin, Scherzer, New, & Garfield, 1971 [68]	13	Prospective	None	Mothers	SB (MMC)	7–11	Late	Psychiatric observation
Kronenberger, 1991(abstract) [69]	66	Prospective	None	Mothers	SB (MMC)	0–18	Early	SCL-90R
Loebig, 1990 [70]	10	Prospective	None	Mothers	SB (MMC)	5–11	Early	Semi-structured interview
McAndrew, 1976 [47]	116	Retrospective	None	Mothers & fathers	Combined: MMC, CP, limb deficit	5–10	-	Semi-structured interview
Murdoch, 1984 [53]	109	Retrospective	None	Mothers	SB	2–10	Early	Semi-structured interview
Nielsen, 1980 [54]	30	Longitudinal	None	Mothers	SB (MMC)	0–6	Early	Semi-structured interview
Richards & McIntosh, 1973 [71]	86	Prospective	None	Mothers & fathers	SB (SBA)	2–6	Late	Semi-structured interview
Rolle, Niemeyer, & Grafe, 2000 [72]	80	Retrospective	None	Mothers & fathers	Combined: SB, HYDRO	0–18	Early	Coping Skills
Spaulding & Morgan, 1986 [59]	19	Prospective	Control	Mothers & fathers	SB-non retarded	5–15	Early	Social Readjustment Rating Scale
Walker, Thomas, & Russell, 1971 [55]	108	Retrospective	None	Mothers & fathers	SB	0–3	Early	Standardized questionnaire

^1^ANC = anencephaly, CP = cerebral palsy, HYDRO = hydrocephalus, MMC = myelomeningocele, NOS = not otherwise specified, NTD = neural tube defect, PKU = phenylketonuria, SB = spina bifida, SBA = spina bifida aperta.

^2^BSI = Brief Symptom Inventory, SCL-90R = Symptom CheckList-90 items Revised.

As shown in Table 1, most studies lacked a comparison group in their design. Only seven studies compared SB-parents with matched control groups, an additional eight studies used standardized outcome measures enabling the comparison of SB-parents with non-clinical norm groups. Most studies included mothers only. Five studies included fathers too, but two of these studies did not specify gender in their analyses [22,23]. Furthermore, some studies included parents of children of all ages whereas others focused on parents of children in a specific developmental period.

Twenty-four reports studied parents of children with SB exclusively. A few studies included late-treated children, that is, children who were born before the time that early surgical treatment had come into practice. Ten studies explicitly confined their samples to the severer forms of SB, namely myelomeningocele (MMC) and spina bifida aperta (SBA). Other studies included a combination of SB with other neural tube defects (NTDs) or with other disabilities. From those non-categorical studies the findings on SB-parents were abstracted for this review. Only one study examined parents' adjustment with the loss of a baby with SB.

Through the years, studies evolved from qualitative to quantitative data collection. Qualitative studies mostly used semi-structured interviews. Quantitative studies used questionnaires to assess symptoms of psychological distress. Three of these measures were adaptations of the Cornell Medical Index, namely: the Malaise Inventory [24], the Symptom Check List-90R (SCL-90R) [25], and the Brief Symptom Inventory (BSI) [26,27]. Other similar questionnaires were the General Health Questionnaire (GHQ) [28] and the Langner Symptom Checklist [29].

#### Step 4: Allocation of studies eligible for meta-analysis

The reviewers selected studies for meta-analysis guided by the following criteria: (1) quantitatively measuring psychological adjustment in samples that include parents of children with SB, (2) including control group scores or using standardized measures for which norm scores are available, (3) reporting sufficient statistics to estimate effect sizes of SB on parents' psychological adjustment and/or to estimate effect sizes of relationships between other factors and parents' psychological adjustment.

Fifteen research reports were eligible for meta-analysis and 18 were not. The reviewers' agreement was 89.8% (Cohen's Kappa = .88). Differences were resolved through discussion.

### Meta-analytic procedures

#### Weighted average effect size d_+_

To estimate the effect of SB on parents' psychological adjustment the weighted average effect size *d*_+ _was calculated [30]. First, one effect size per sample was obtained through combining multiple reports on the same sample to avoid overrepresentation [31-34]. Second, for studies using standardized outcome measures without matched control groups, Malaise Inventory scores of SB-mothers were compared with norm scores of 33-year-old women (*N *= 5678, *M *= 2.81, *SD *= 3.18; physical health *M *= .89, *SD *= 1.17; mental health *M *= 1.89, *SD *= 2.37) of the National Child Development Study [35]; scores on the Symptom Check List-90 Revised Global Severity Index were compared with the adult non-patient scores of women (*N *= 480, *M *= .36, *SD *= .35 or *T *= 50, *SD *= 10) and of men (*N *= 494, *M *= .25, *SD *= .24 or *T *= 50, *SD *= 10) [25]; and *T*-scores on the Brief Symptom Inventory Global Severity Index were compared with the norms for women (*N *= 480, *T *= 50, *SD *= 10) and men (*N *= 494, *T *= 50, *SD *= 10). Third, the statistical program SISA Binomial [36] was used to estimate a corrected number of degrees of freedom in cases where experimental and comparison groups had different variances. Fourth, effect sizes *g *were calculated based on means and standard deviations or based on *t*-test scores [30,37]. Fifth, *g*'s were converted into *d*'s correcting for bias because the reports in this review had relatively small samples. Finally, the weighted average *d*_+ _was calculated [30]. For all *d*_+_'sStouffer's combined probability effect sizes *Z*_*c *_were reported as indicators of significance.

To check whether *d*_+ _encompassed zero, a 95% confidence interval (CI 95%) was estimated. The actual magnitude of *d*_+ _was interpreted through use of Cohen's [38] guidelines: *d*_+ _≤ .2 (small effect), *d*_+ _≤ .5 (medium effect), and *d*_+ _≤ .8 (large effect). Furthermore, *d*_+_'s were transformed into percentiles of the normal distribution (*U*_*3*_) using Cohen's [38] table to study the amount of non-overlap between experimental and comparison groups. Finally, the homogeneity statistic *Q *[30] was calculated to determine whether the set of *d*'s on which *d*_+ _was based shared a common effect.

Moderating effects of study or sample characteristics on *d*_+ _were not tested because of the small number of studies (*k *= 15).

#### Weighted average effect size r

To estimate associations between parents' psychological adjustment and various factors the weighted average effect size *r *[39] was computed. First, *t*-test and *F-*test estimates were converted into Pearson's correlations. Second, raw correlation coefficients *r *were transformed into Fisher's *Z*_*r *_allowing the sampling distribution of *r *to approximate a Gauss curve. Third, each *Z*_*r *_was weighted by the reciprocal of its estimated within-study variance [30]. Combined probability levels *Z*_*c *_were obtained through dividing the average effect sizes *Z*_*r *_by their standard errors.

Regarding the interpretation of *r*, most authors recognize that a minimum of three studies is needed for *r *to be a valid estimate of the population effect size *Rho *[37]. However, since the objective of this review was to exhaust the limited studies available as much as possible, *r*'s were also calculated on two correlation coefficients. Our justification is that any significant correlation expresses a representative estimate of an association in a certain population. Thus, although two combined correlations do not sufficiently approximate effect size *Rho *of the universal population, they do at least indicate a valid association in two independent populations.

The *r*'s based on three or more correlation coefficients were interpreted as follows. The magnitude of *r *was interpreted using Cohen's [38] guidelines: *r *= .1 (small effect), *r *= .3 (medium effect), and *r *= .5 (large effect). Furthermore, the *Q *statistic was computed to test the homogeneity of studies underlying *r*.

#### File drawer analysis Fail Safe N

Reviews based on published studies only, may be at risk for Type I errors. The underlying assumption is that studies revealing nonsignificant results (confirming the null-hypothesis) are less likely to be published than studies reporting significant results. One way to correct for such bias is to calculate the number of studies confirming the null-hypothesis that would be necessary to reverse a conclusion that a significant relationship exists [37]. Because unpublished manuscripts were beyond the scope of this review, both meta-analyses were followed by File Drawer Analysis [40,41]. In this review, the Fail Safe *N *[42] was calculated.

## Results

### Weighted average effect size d_+_

The first question was whether parents of children with SB showed higher levels of psychological distress than comparison groups. The group means, standard deviations, *t*-tests, raw group differences, and Hedges' standardized effect sizes *g *and *d *of SB-parents and comparison groups are displayed in an additional file [see Additional file 1]. Based on these data, *d*_+_'s were computed. Table 2 presents the statistics *d*_+_, C.I. 95%, Cohen's *U*_*3*_, homogeneity test *Q*, Stouffer's combined *Z*_*c*_, and Fail Safe .05 *N *for mothers, fathers, and parents.

**Table 2:** Weighted average effect sizes of SB on parents' psychological adjustment

	***k***	***n*_*exp*_**	**Weighted Mean** **Effect Size *d*_+_**	**95% Confidence Interval**	***U*_*3*_**	**Homogeneity Test *Q***	**Stouffer's Combined Test *Z*_*c*_**	**Fail Safe .05 *N***
Mothers	10	500	.73	.38 – .97	76.7%	66.21***	9.15***	299.1
Fathers	3	134	.54	.35 – .76	70.5%	0.24	3.93***	14.1
Parents	15	831	.76	.48 – .86	77.6%	73.54***	11.25***	686.7

*** *p *< .0001

For mothers of children with SB the average amount of psychological distress was .73 standard deviations higher than for controls (see Table 2). This effect size was between medium and large. The C.I. 95% did not include zero, indicating that the chance of not finding a negative effect of SB was less than 5%. Furthermore, there was 76.7% of non-overlap between SB-mothers and comparison groups. The summary index of statistical significance (*Z*_*c*_) further underscored the probability of the effect. Finally, the Fail Safe *N *revealed that more than 299 studies confirming a null-hypothesis would be needed to overturn the effect.

For fathers of children with SB more moderate though similar findings were obtained based on three studies. Their levels of psychological distress were approximately half a standard deviation higher than the comparison groups, indicating a medium to large effect size. The corresponding non-overlap between the groups was 70.5%. The C.I. 95% and *Z*_*c *_indicated that the effect was consistent and significant. Moreover, 14 nonsignificant studies would be needed to reverse the effect.

For all parents taken together a medium to large negative effect (*d*_+ _= .76) of SB psychological adjustment was found. There was 77.6% non-overlap between SB-parents and comparison groups. The C.I. 95% did not include zero and *Z*_*c *_confirmed the overall significance of the effect. What is more, 687 studies confirming a null-hypothesis would be required to undermine the effect size.

Notwithstanding the above results, the significance of *Q *indicated that the effects of SB on mothers' psychological functioning varied greatly. For fathers a homogeneous underlying effect size was confirmed by a nonsignificant *Q *but the number of studies (*k = 3*) was rather limited.

### Weighted average effect size r

Possible explanations for the heterogeneity of the SB effect on parents' psychological adjustment were studied by examining associated factors. The variables studied in relationship with parents' psychological functioning had been categorized as: child factors, parent factors, family factors and environmental factors. A summary of the converted effect sizes (Pearson's *r*, *p*-value, and Fisher *Z*_*r*_) found in the literature is displayed in an additional file [see Additional file 2]. The weighted average effect sizes *r *are depicted in Table 3. In the following, only the results for the average effect sizes *r *based on three or more studies will be briefly described.

**Table 3:** Weighted average effect sizes of categories associated with parents' psychological symptoms

**Category**	***k***	***n***	**Weighted *Z*_*r*_**	**Effect Size *r***	***Z*_*c*_**	**Homogeneity *Q***	**Fail Safe .05 *N***
*Child factors*							
Disability parameters	4	385	.14	.14	2.75**	2.93	10.1
Behavior problems	3	273	.38	.37	6.22***	2.60	30.9
Emotional problems	2	193	.50	.47	6.90***	1.03	30.4
Social competence	2	109	-.12	-.12	-1.26	.02	0.0
							
*Parent factors*							
Socio-economic characteristics	3	264	-.13	-.13	-2.13*	1.45	.36
Appraised stress	2	177	.56	.63	7.32***	5.90*	30.8
Coping	2	76	.40	.38	3.31***	8.55**	10.9
Parenting satisfaction/competence	2	109	-.44	-.41	-4.44***	.09	12.1
							
*Family factors*							
Partner presence	3	211	-.16	-.16	-2.22*	.69	1.6
Marital adjustment	2	97	-.43	-.40	-4.12***	.23	10.4
Family income	2	214	-.22	-.22	-3.15**	1.05	6.2
Positive family environment	5	340	-.45	-.42	-8.14***	1.17	108.6
							
*Environment factors*							
Quantity social support	4	240	-.29	-.28	-4.35***	3.16	22.9
Satisfaction social support	4	351	-.29	-.28	-5.37***	6.68	37.9
Formal support	2	214	-.07	-.07	-1.07	.01	.0

* *p *< .05, ** *p *< .01, *** *p *< .001

Seven child variables were reported in association with parents' psychological adjustment: gender, age, cognitive capacities, disability parameters, behavior problems, emotional problems, and social competence [see Additional file 2]. Disability parameters had a small positive and behavior problems had a medium positive association with parents' psychological symptoms (see Table 3). Both effects were homogeneous.

Five parent variables were studied in relation to parental adjustment: socio-economic characteristics, appraised stress, hope, coping, and parenting satisfaction-competence [see Additional file 2]. Socio-economic characteristics correlated inversely and very minimally to parents' psychological complaints (see Table 3). The significance level that was reached mainly reflected correlations found by Kronenberger and Thompson [20].

Eight family variables were studied in association with parental adjustment: partner presence, marital adjustment, family income, family size, family coping style, impact on family, negative family environment, and positive family environment [see Additional file 2]. The presence of a partner was correlated with fewer psychological symptoms, albeit minimally (see Table 3). Moreover, two nonsignificant studies would be enough to nullify the association. Positive family environment was moderately but consistently related with less psychological complaints. Both *r*'s were homogeneous.

Three environmental factors were reported in connection with parents' adjustment: Quantity of social support, social support satisfaction and formal support [see Additional file 2]. For both the amount of social support and satisfaction with social support medium effects were found on psychological distress (see Table 3). The Fail Safe *N *indicated that the effects would not be easily overturned. Both *r*'s were homogeneous.

## Discussion

### Overall results

In this section the meaning of the above findings will be addressed and specific gaps in our understanding of parental psychological adjustment with SB will be identified.

#### Levels of psychological distress in parents of children with SB

The results confirmed our hypothesis that the presence of SB in families predicts higher levels of psychological strain in parents. The heterogeneity of the effect for mothers however also indicated that SB does not necessarily provoke psychopathology in all parents. Reports on the proportions of SB-parents, scoring within clinical ranges of psychopathology, further illustrate this. Within samples of SB-mothers varying proportions of psychopathology were found: 19.2% [43], 31.9% [44], 41% [45], 50% [46] and 56% [47]. Less variability was found for SB-fathers: 25.6% [43], and 28% [48].

#### Gender differences in parents' psychological adjustment

It was hypothesized that differences in the effect of SB on adjustment could be expected between mothers and fathers because of role differentiations in care and work. The effect for mothers seemed somewhat higher than for fathers, but the difference could not be tested reliably because of the few studies on fathers. There was some indication that the effect of SB was more homogeneous for fathers than for mothers. Hypothetically, the division of care and work between partners may provide a theoretical explanation for this difference. Work outside the home can be an opportunity to release some of the stress around SB [49]. While at the same time, full-time working schedules may impede contacts with health professionals and therefore diminish opportunities to discuss worries concerning SB [48]. Fathers tend to work fulltime schedules while mothers' occupational lives are more likely to vary [48,49]. In addition, the nursing burden for children with SB varies greatly. Thus SB-related stress on mothers may be much more variable than on fathers. Further enquiries on father's psychological adjustment with SB will be needed to determine whether this hypothesis can be empirically underscored.

#### Factors correlated with parents' psychological adjustment

Variability in parents' psychological adjustment was expected to be associated with child, parent, family, and environment factors. In terms of models explaining adjustment to chronic illness, parents' psychological adjustment was regarded as the outcome of transactions among multiple factors representing demands and resources. Theoretically, such transactions may involve interactions as well as main effects; however in this meta-analysis direct associations were estimated only.

**Figure 1:**
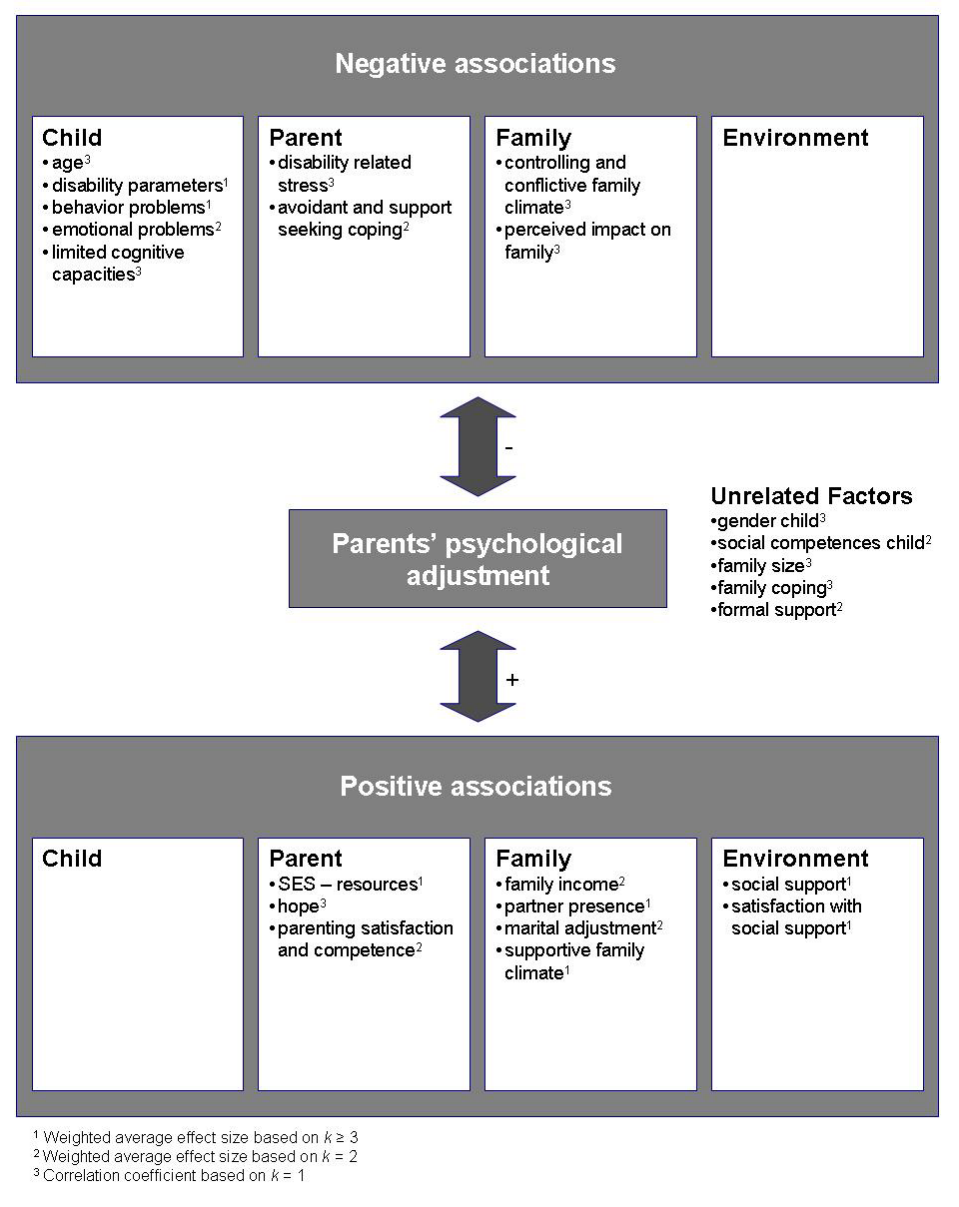
Factors found to be associated with parents' psychological adjustment.

This review yielded correlation coefficients based on one study only (Additional file 2; representative of one population), average effect sizes based on two studies (Table 3; representative of two populations), and average effect sizes based on three or more studies (Table 3; representative of all populations). Figure 1 displays a summary of these associations.

All the associations were cross-sectional. Hence, inferences about causalities, whether uni- or bi-directional, could not be made on an empirical basis. In the light of this situation, it is feasible that future longitudinal studies will reveal that not all of the associations found in this review will be of decisive importance to explain parents' adjustment with SB. Therefore, we labeled factors associated with reductions in psychological distress as "positive associations" and factors associated with increases in parents' psychological distress as "negative associations".

#### Child factors

Associations of the child's cognitive capacities with parents' psychological adjustment were hardly reported, despite indications from non-categorical studies that cognitive limitations are likely to put extra strains on parents [17] and despite indications from neuropsychological research that children with SB have specific profiles of cognitive strengths and weaknesses [50]. More research will be needed to understand the impact of children's cognitive profiles on parents' adjustment.

Most studies did not find associations between the degree of the physical disability and parents' psychological adjustment, except one study [34]. Kronenberger and Thompson [21] have noted that this particular study included children with milder forms of SB. Another explanation may be that indexes of the severity of SB have not been conceptualized in a consistent way. Some studies used indicators of physical impairments only (e.g., lesion level of the defect), others added functional limitations (e.g., the degree of mobility), and/or indicators of treatment intensity (e.g., number of shunt revisions). Conceptual refinement of SB-parameters and treatment will be needed to more effectively investigate which factors cause stress in parents and which do not.

Theoretically, the marginality hypothesis [16] may further explain why a linear relationship between SB parameters and parents' psychological functioning was barely found. This theory holds that children with minor disabilities tend to exhibit more psychosocial problems than severely impaired children because they have difficulties identifying themselves with either able-bodied or disabled peers. Similar identification problems could arise for parents of marginally disabled children with SB.

The associations of behavior and emotional problems with parents' psychological symptoms may signify that such problems put additional strain on parents. Once children have developed conduct and/or emotional disorders, this line of reasoning is plausible. However, it is well known from the parenting literature that parent-child relationships are bidirectional, meaning that parents and children mutually influence each other through long-term transactions. For example, attachment theorists have emphasized that during the early years of the child's life, parents' sudden mood changes, depressive symptoms, and grief are potential risks to the development of affective attunement between parent and child [51]. Parents descriptions in open interviews of their struggle with emotions during the first year after the birth of their child with SB seem to support the hypothesis that these children might be at risk of insecure attachment [47,52-55]. In the long term, the insecure parent-child relationship may contribute to the development of behavioral and emotional problems. It may be well worth studying the early development of parent-child relations in families with SB to uncover how children's behavioral and emotional problems interplay with parents' psychological adjustment over time.

Children's lack of social competence was not found to be related to parents' psychological health even though Lemanek et al. [56] reported that children with SB had significantly fewer social skills than children in norm groups. These findings provide indirect support for the hypothesis that parents do not expect equal proficiency in social skills, such as cooperation, assertion, responsibility, and self-control, from a child with SB as from an able-bodied child. This may explain why a limitation in social skills of children with SB does not affect parents' psychological adjustment. More studies investigating parents expectations are required to affirm this assumption.

#### Parent factors

Very few studies investigated the role of parents' appraisals and coping styles. This is remarkable, since the role of appraisal and coping are of central importance to understanding how stressful events affect people [57]. The scarce findings suggest that parents' appraisals (e.g., appraised stress and hope) and coping styles are highly predictive of positive as well as negative adjustment. Besides appraisal and coping, hardly any intra-personal resources of parents were studied in relation with psychological adjustment to SB. In the light of current theories on affect-processing, the absence of studying personality characteristics, such as ego-resilience, could be regarded a major gap in our knowledge of parents' adjustment to SB. For example, J. Block has pointed out that some individuals are characteristically maladaptive while others are characteristically resourceful in responding to environmental stressors [58]. This characteristic ability to dynamically and progressively adapt to stress appears to be more person-related than situation-related. Thus, studies on the associations between personality characteristics, affect-regulation, and psychological adjustment may prove to be fruitful.

#### Family factors

As expected, parents' psychological health was consistently associated with a supportive family climate. The quality of parents' partner relationship also appeared to be a promising correlate of psychological bonadjustment. Future research may need to study more closely though, whether the measure of marital satisfaction reflects satisfaction with the joint care for the child with SB or satisfaction with a relationship that meets parents' personal needs of intimacy and companionship.

#### Environmental factors

In line with expectations, there appears to be fair evidence that a large informal social network of family and friends that matches parents' needs, enhances parents' psychological adjustment to SB. Unexpectedly, formal types of support were not related to parents' psychological adjustment. Apparently, dissatisfaction with formal support does not necessarily imply increased risks of psychological maladjustment.

### Strengths and limitations of studies and future research

The chronology of studies was in line with contemporary trends in behavioral sciences. Qualitative descriptive research was followed by quantitative analytical designs. Standardized measures of psychological symptoms came into use and were updated, passing from the Malaise Inventory to the Brief Symptom Inventory. Statistical procedures moved from correlational analyses to multiple regression equations and structural equation models.

Inevitably, studies also had methodological limitations. In the first place, studies had sampling problems. Samples tended to be small, risking Type II errors (i.e. not detecting a relationship which in fact exists). For example, one study (*n *= 19) did not find a significant relationship between SB and parents' psychological adjustment [59]. Furthermore, the recruitment of participants via hospitals and/or SB associations may have led to unbalanced sampling. Members of SB associations may not be representative of all SB-parents. Moreover, parents with psychiatric problems may have refused to participate in studies. And finally, fathers were underrepresented.

A second area of concern is the quality of the associations reported in studies. Most associations were cross-sectional. Hence, the causal interpretations were based on theoretical assumptions only. Furthermore, correlations may have been inflated because studies relied on parents' self-reports. Especially studies examining depression and anxiety are at risk of common method variance, because the respondents' affective states may influence their ratings of other concepts [60].

Future studies will need to increase their sample sizes through merging datasets from different studies and establishing long-term multi-centered collaborations. Special efforts, such as home visits after office hours, must be made to include more fathers. Longitudinal designs are needed to empirically validate assumed directions of associations. And finally, studies need to collect data from multiple informants and/or observational data to avoid common method variance.

## Conclusion

Our study confirms that SB represents a considerable challenge to parents' psychological well-being. Especially mothers are at risk of psychological suffering, although there is great variety among mothers in their psychological adjustment to having a child with SB. Studies indicate that the extent to which SB affects parents depends on the quality of parents' partner relationship, family climate, and support from informal social networks.

### Clinical implications

Bearing these results in mind, it is important to monitor parents' psychological well-being on a regular basis, that is, to ask parents at different stages of their child's life how they cope, how they keep the care strains manageable, how they support one another, and how they reserve time to balance the care for their child with SB and other primary tasks with their personal needs. Alertness to the quality and amount of social support around the family may prevent parents from becoming overburdened.

It may be important to advise parents to think strategically about how their relationships with others can support them emotionally as well as instrumentally at times when the care for their child intensifies due to acute medical situations or at times when chronic burdens pile up. At the same time, it may be equally important to advise parents to think about how much attention these relationships need in order to be maintained.

In conclusion, the medium-large effect of SB on parents' psychological health indicates that spina bifida health care should include psychological support to parents of children with this condition to ensure the well-being of the whole family.

## Competing interests

The author(s) declare that they have no competing interests.

## Authors' contributions

IV conceived of the study, carried out the literature search, the meta-analyses, and drafted the manuscript. JJ helped with the literature search (coding and categorizing abstracts), supervised the meta-analyses procedures, and helped to draft the manuscript. AB participated in the interpretation of the findings and critically revised the content of the manuscript. JG conceived of the study, supervised its design and coordination, and critically revised the content of the manuscript.

## Pre-publication history

The pre-publication history for this paper can be accessed here:



## Supplementary Material

Additional File 1 Effect sizes of SB on parents' psychological adjustment. This file contains a table with the statistics that we gathered from primary reports. Based on these statistics the weighted average effect sizes of spina bifida on parents' psychological adjustment were estimated. Click here for file

Additional File 2 Reported correlations of factors associated with parents' psychological adjustment. This file contains a summary of the converted effect sizes (Pearson's r, p-value, and Fisher Zr) found in the literature. Based on these data the weighted average effect sizes of categories associated with parents' psychological symptoms were estimated. Click here for file
