# Supplementary material for: Parents' psychological adjustment in families of children with Spina Bifida: a meta-analysis
Source: BMC Pediatr. 2005 Aug 25;5:32. doi: 10.1186/1471-2431-5-32 (PMC1215488; doi:10.1186/1471-2431-5-32)
Supplement: Additional File 1 — Effect sizes of SB on parents' psychological adjustment. This file contains a table with the statistics that we gathered from primary reports. Based on these statistics the weighted average effect sizes of spina bifida on parents' psychological adjustment were estimated. [file 1471-2431-5-32-S1.doc]

Additional file 1 - Effect sizes of SB on parents’ psychological adjustment

| Study | | Experimental group | | | Control group | | | Raw difference | | | | | Standardized E.S. | |
| --- | --- | --- | --- | --- | --- | --- | --- | --- | --- | --- | --- | --- | --- | --- |
| Authors | Outcome measure | *M* | *SD* | *n* | *M* | *SD* | *n* | Pooled *SD* | *M_experimental_ -M_control_* | Student *t*-test | *p*-value for difference in Means (one-tailed) | Corrected *Df* | Hedges’ E.S. based on pooled *SD*s (*g*) | Hedges’ E.S. corrected for bias (*d*) |
| **Mothers** |  |  |  |  |  |  |  |  |  |  |  |  |  |  |
| Tew & Laurence, 1973 | Malaise Inventory | 6.08 | 5.26 | 51 | 2.81 | 3.18 | 5678 | 3.20 | 3.27 | 4.43 | 2.7E-4 | 50 | 1.02 | 1.02 |
| Wallander et al., 1989 | Malaise Inventory physical health | 2.66 | 2.08 | 50 | .89 | 1.17 | 5678 | 1.18 | 1.77 | 6.01 | 1.0E-5 | 49 | 1.50 | 1.50 |
| Wallander et al., 1989 | Malaise Inventory mental health | 2.84 | 2.42 | 50 | 1.89 | 2.37 | 5678 | 2.37 | .95 | 2.82 | 3.9E-3 | 49 | .40 | .40 |
| Fagan & Schor, 1993 | Malaise Inventory | 6.20 | 4.30 | 50 | 2.81 | 3.18 | 5678 | 3.19 | 3.39 | 5.56 | 1.0E-5 | 49 | 1.06 | 1.06 |
| Kazak & Marvin, 1984 | Langner Symptom Checklist | 3.80 | -- | 56 | 1.98 | -- | 53 | -- | 1.82 | 2.59 | .025 | 101 | .52^1^ | .51 |
| Kronenberger & Thompson, 1992a, 1992b | SCL-90R GSI  (T-scores) | 56.50 | 11.40 | 66 | 50.00 | 10.00 | 480 | 10.18 | 6.50 | 4.86 | 1.5E-5 | 79 | .64 | .64 |
| Holmbeck et al., 1997 | SCL-90R GSI  (raw scores) | .51 | .39 | 55 | .37 | .33 | 55 | .36 | .14 | 2.03 | .022 | 105 | .39 | .38 |
| Barakat & Linney, 1992 | BSI GSI  (raw scores) | 1.88 | .13 | 29 | 1.84 | .15 | 28 | .14 | .04 | 1.08 | .142 | 53 | .29 | .28 |
| Wiegner & Donders, 2000 | BSI GSI  (T-scores) | 57.68 | 10.93 | 34 | 50.00 | 10.00 | 480 | 10.06 | 7.68 | 4.30 | 1.6E-4 | 37 | .76 | .76 |
| Lemanek et al., 2000 | SCL-90R GSI | 52.12 | 10.56 | 59 | 50.00 | 10.00 | 480 | 10.06 | 2.12 | 1.53 | .073 | 71 | .21 | .21 |

| Study | | Experimental group | | | Control group | | | Raw difference | | | | | Standardized E.S. | |
| --- | --- | --- | --- | --- | --- | --- | --- | --- | --- | --- | --- | --- | --- | --- |
| Authors | Outcome measure | *M* | *SD* | *n* | *M* | *SD* | *n* | Pooled *SD* | *M_experimental_ -M_control_* | Student *t*-test | *p*-value for difference in Means (one-tailed) | Corrected *DF* | Hedges’ E.S. based on pooled *SD*s (*g*) | Hedges’ E.S. corrected for bias (*d*) |
| **Fathers** |  |  |  |  |  |  |  |  |  |  |  |  |  |  |
| Kazak & Marvin, 1984 | Langner Symptom Checklist | 2.56 | -- | 42 | 1.91 | -- | 30 | -- | .65 | 2.59 | .025 | 70 | .62^1^ | .61 |
| Evans, Tew, & Laurence, 1986 | GHQ | 4.30 | -- | 49 | 1.10 | -- | 46 | -- | 3.20 | 2.81 | .003 | 93 | .58^1^ | .57 |
| Holmbeck et al., 1997 | SCL-90R GSI  (raw scores) | .39 | .33 | 43 | .26 | .22 | 43 | .28 | .13 | 2.15 | .018 | 73 | .46 | .46 |
| **Parents** |  |  |  |  |  |  |  |  |  |  |  |  |  |  |
| King et al., 1999 | SCL-90R GSI  (raw scores) | .64 | .55 | 164 | .31 | .31 | 974 | .35 | .33 | 7.49 | 1.0E-5 | 180 | .93 | .93 |
| Venters-Horton & Wallander, 2001 | BSI GSI  (T-scores) | 57.68 | 10.93 | 33 | 50.00 | 10.00 | 974 | 10.03 | 7.68 | 4.33 | 1.7E-4 | 33 | .77 | .77 |

^1^ *g* was calculated from *t* and *df*; *g = 2t/√df*.

^2^ Wallander et al. (1989) reported the means and standard deviations separately for the subscales mental and physical complaints of the Malaise Inventory. For the estimation of *g* and d we maintained this separation however the corresponding weights were corrected and divided in half to prevent disproportional weighting of the Wallander study in the calculation of the average *d_+_*.
