# Supplementary material for: Parents' psychological adjustment in families of children with Spina Bifida: a meta-analysis
Source: BMC Pediatr. 2005 Aug 25;5:32. doi: 10.1186/1471-2431-5-32 (PMC1215488; doi:10.1186/1471-2431-5-32)
Supplement: Additional File 2 — Reported correlations of factors associated with parents' psychological adjustment. This file contains a summary of the converted effect sizes (Pearson's r, p-value, and Fisher Zr) found in the literature. Based on these data the weighted average effect sizes of categories associated with parents' psychological symptoms were estimated. [file 1471-2431-5-32-S2.doc]

**Additional file 2 – Reported correlations of factors associated with parents’ psychological a**djustment

| **Study** | **Factors** | | **Psychological**  **Symptoms^3^** | ***n*** | ***r*** | **One-tailed**  ***p*** | **Fisher’s**  ***Z_r_*** |
| --- | --- | --- | --- | --- | --- | --- | --- |
|  | **Child categories** | **Child variables** |  |  |  |  |  |
| Lemanek, et al., 2000 | Gender | Gender | SCL-90R | 59 | .09 | .249 | **.09** |
| Lemanek, et al., 2000 | Age | Age | SCL-90R | 59 | .22 | .047 | **.22** |
| Tew & Laurence, 1975^1^ | Cognitive capacities | IQ (< 80 vs 80+) | Mal Inv | 51 | -.45 | 4.6E-4 | -.48 |
| Tew & Laurence, 1975^1^ |  | Education (regular vs special) | Mal Inv | 51 | -.46 | 3.4E-4 | -.50 |
| King, et al., 1999 | Disability parameters | Health problems | SCL-90R | 164 | .02 | .400 | .02 |
| King, et al., 1999 |  | Functional dependence | SCL-90R | 164 | .07 | .187 | .07 |
| King, et al., 1999 |  | Need of services | SCL-90R | 164 | .12 | .063 | .12 |
| Kronenberger & Thompson, 1992b |  | Lesion level | SCL-90R | 66 | .01 | .468 | .01 |
| Kronenberger & Thompson, 1992b |  | Presence of shunt | SCL-90R | 66 | -.02 | .437 | -.02 |
| Kronenberger & Thompson, 1992b |  | Child age when shunted | SCL-90R | 66 | .04 | .375 | .04 |
| Kronenberger & Thompson, 1992b |  | Nr of shunt operations | SCL-90R | 66 | .10 | .212 | .10 |
| Kronenberger & Thompson, 1992b |  | Time since last shunt revision | SCL-90R | 66 | .11 | .190 | .11 |
| Kronenberger & Thompson, 1992b |  | Chiari operation | SCL-90R | 66 | -.04 | .375 | -.04 |
| Lemanek, et al., 2000 |  | Severity of impairment | SCL-90R | 59 | .11 | .203 | .11 |
| Tew & Laurence, 1975^1^ |  | Severity of lesion | Mal Inv | 51 | .38 | .003 | .40 |
| Tew & Laurence, 1975^1^ |  | Severity of incontinence | Mal Inv | 51 | .32 | .011 | .33 |
| Tew & Laurence, 1975^1^ |  | Severity of impairment | Mal Inv | 51 | .28 | .023 | .29 |
| Venters & Wallander, 2001 |  | Severity physical disability | BSI | 111 | .18 | .029 | .18 |
| King, et al., 1999 | Behavior problems | Conduct disorder | SCL-90R | 164 | .37 | 5.4E-7 | .39 |
| King, et al., 1999 |  | Hyperactivity disorder | SCL-90R | 164 | .43 | 4.5E-9 | .46 |
| Lemanek, et al., 2000 |  | Behavior problems | SCL-90R | 59 | .41 | .001 | .44 |
| Wallander, et al., 1989^2^ |  | Behavior problems | Mal Inv | 50 | .17 | .119 | .17 |
| Barakat & Linney, 1992 | Emotional problems | Internalizing behavior | BSI | 29 | .60 | 2.8E-4 | .69 |
| King, et al., 1999 |  | Emotional disorder | SCL-90R | 164 | .50 | 4.6E-12 | .55 |
| King, et al., 1999 |  | Somatization | SCL-90R | 164 | .38 | 2.6E-7 | .40 |
| Lemanek, et al., 2000  Wallander, et al., 1989^2^ | Social competence | Social skills  Social competence | SCL-90R  Mal Inv | 59  50 | -.11  -.13 | .203  .184 | -.11  -.14 |
|  | **Parent categories** | **Parent variables** |  |  |  |  |  |
| Fagan & Schor, 1993 | Socio-economic char. | Mother’s race | Mal Inv | 50 | .01 | .473 | .01 |
| King, et al., 1999 |  | Parent’s educational level | SCL-90R | 164 | -.15 | .028 | -.15 |
| King, et al., 1999 |  | Parent’s employment | SCL-90R | 164 | -.22 | .002 | -.22 |
| Wallander, et al., 1989^2^ |  | Mother’s SES-age | Mal Inv | 50 | -.08 | .290 | -.10 |
| Kronenberger & Thompson, 1992b | Appraised stress | Child-related medical stress | SCL-90R | 66 | .35 | .002 | .37 |
| Kronenberger & Thompson, 1992b |  | Social-nonchild stress | SCL-90R | 66 | .25 | .021 | .26 |
| Venters & Wallander, 2001 |  | Disability-related stress | BSI | 111 | .60 | 1.6E-12 | .70 |
| Venters & Wallander, 2001 | Hope | Hope | BSI | 111 | -.38 | 1.9E-5 | **-.40** |
| Barakat & Linney, 1995 | Coping | Avoidant coping | BSI | 29 | .69 | 1.6E-5 | .85 |
| Kronenberger & Thompson, 1992a |  | Seeking support of family | SCL-90R | 47 | .17 | .127 | .17 |
| Kronenberger & Thompson, 1992a |  | Seeking support of spouse | SCL-90R | 47 | -.20 | .089 | -.20 |
| Kronenberger & Thompson, 1992a |  | Seeking support of friends | SCL-90R | 47 | .39 | .003 | .41 |
| Fagan & Schor, 1993  Fagan & Schor, 1993 | Parenting satisfaction and competence | Parenting competence  Parenting satisfaction | Mal Inv  Mal Inv | 50  50 | -.45  -.32 | 5.1E-4  .012 | -.48  -.33 |
| Lemanek, et al., 2000  Lemanek, et al., 2000 |  | Parenting efficacy  Parenting satisfaction | SCL-90R  SCL-90R | 59  59 | -.26  -.58 | .023  7.1E-7 | -.27  -.66 |
|  | **Family categories** | **Family variables** |  |  |  |  |  |
| Fagan & Schor, 1993 | Partner presence | Presence adult companion | Mal Inv | 50 | -.08 | .290 | -.08 |
| Venters & Wallander, 2001 |  | Marital stat (single vs married) | BSI | 111 | -.21 | .013 | -.21 |
| Wallander, et al., 1989^2^ |  | Duration of marriage | Mal Inv | 50 | -.11 | .224 | -.11 |
| Wallander, et al., 1989^2^ | Family size | Family structure | Mal Inv | 50 | .15 | .149 | **.15** |
| Kronenberger & Thompson, 1992a  Wallander, et al., 1989^2^ | Marital adjustment | Marital adjustment  Marital adjustment | SCL-90R  Mal Inv | 47  50 | -.36  -.45 | .006  5.1E-4 | -.38  -.48 |
| Fagan & Schor, 1993  King et al., 1999 | Family income | Family income  Family income | Mal Inv  SCL-90R | 50  164 | -.34  -.18 | .008  .011 | -.35  -.18 |
| Fagan & Schor, 1993  King, et al., 1999 | Positive family environment | Family functioning  Family functioning | Mal Inv  SCL-90R | 50  164 | -.47  -.38 | 2.8E-4  2.6E-7 | -.51  -.40 |
| Barakat & Linney, 1992  Kronenberger & Thompson, 1992a  Wallander, et al., 1989^2^ |  | Family support  Family support  Family support | BSI  SCL-90R  Mal Inv | 29  47  50 | -.46  -.51  -.41 | .006  1.2E-4  .002 | -.50  -.56  -.44 |
| Kronenberger & Thompson, 1992a Kronenberger & Thompson, 1992a | Negative family environment | Family conflict  Family control | SCL-90R SCL-90R | 47  47 | .39  .37 | .003  .005 | .41  .39 |
| King, et al., 1999 | Family coping style | Family coping style | SCL-90R | 164 | -.05 | .262 | **-.05** |
| King, et al., 1999 | Impact on family | Impact on family | SCL-90R | 164 | .22 | .003 | **.22** |
|  | **Environment cat.** | **Environment variables** |  |  |  |  |  |
| Barakat & Linney, 1992  Fagan & Schor, 1993  Venters & Wallander, 2001  Wallander, et al., 1989^2^ | Quantity of social support | Available social network  Social support  Social support number  Social network support | BSI  Mal Inv  BSI  Mal Inv | 29  50  111  50 | -.49  -.18  -.32  -.16 | .003  .105  3.1E-4  .134 | -.54  -.18  -.33  -.16 |
| Barakat & Linney, 1992  King, et al., 1999  Kronenberger & Thompson, 1992a  Venters & Wallander, 2001 | Social support satisfaction | Social support satisfaction  Social support satisfaction  Social support satisfaction  Social support satisfaction | BSI  SCL-90R  SCL-90R  BSI | 29  164  47  111 | -.41  -.17  -.21  -.44 | .013  .015  .078  6.7E-7 | -.44  -.17  -.21  -.47 |
| King, et al., 1999 | Formal support | Rehabilitation centre satisfaction | SCL-90R | 164 | -.06 | .223 | -.06 |
| King, et al., 1999 |  | Health care: information | SCL-90R | 164 | -.03 | .351 | -.03 |
| King, et al., 1999 |  | Health care: support | SCL-90R | 164 | -.11 | .080 | -.11 |
| Wallander, et al., 1989^2^ |  | Service utilization | Mal Inv | 50 | -.08 | .290 | -.09 |

^1^ Values represent Pearson correlations *r* transformed directly from *t*.

^2^ Correlations of factors with the subscales mental and physical health problems (Malaise Inventory) were combined into one correlation with psychological symptoms.

^3^ BSI = Brief Symptom Inventory, Mal Inv = Malaise Inventory, SCL-90R = Symptom Check List – 90 Revised.
